# Snail1: A Transcriptional Factor Controlled at Multiple Levels

**DOI:** 10.3390/jcm8060757

**Published:** 2019-05-28

**Authors:** Josep Baulida, Víctor M. Díaz, Antonio García de Herreros

**Affiliations:** 1 Programa de Recerca en Càncer, Institut Hospital del Mar d’Investigacions Mèdiques (IMIM), Unidad Asociada al CSIC, 08003 Barcelona, Spain; 2 Departament de Ciències Experimentals i de la Salut, Universitat Pompeu Fabra, 08003 Barcelona, Spain

**Keywords:** Snail1, transcriptional factor, Epithelial to mesenchymal transition (EMT), tumor invasion, drug resistance

## Abstract

Snail1 transcriptional factor plays a key role in the control of epithelial to mesenchymal transition and fibroblast activation. As a consequence, Snail1 expression and function is regulated at multiple levels from gene transcription to protein modifications, affecting its interaction with specific cofactors. In this review, we describe the different elements that control Snail1 expression and its activity both as transcriptional repressor or activator.

## 1. Introduction

Epithelial to mesenchymal transition (EMT) is a progressive and reversible process that promotes epithelial cells to acquire a mesenchymal phenotype. During this transition, the cell-cell junction structures, including adherens junctions and desmosomes, are disassembled. Cells lose their cobblestone appearance and adopt a spindle-shaped morphology. EMT provides epithelial cells with different traits relevant for tumorigenesis since, upon EMT, cells become more motile and invasive, become more resistant to pro-apoptotic stimuli, reprogram their metabolism, and acquire characteristics of cancer stem cells. For these reasons, EMT has attracted the attention of many cancer biologists and has been extensively studied in recent years. Many excellent and recent reviews have addressed different insights in EMT and the acquisition of high-grade malignancy [1,2,3].

EMT is orchestrated by a set of EMT-activating transcriptional factors (EMT-TFs), whose core set includes Snail1 (Snail), Snail2 (Slug), Twist1, Zeb1, and Zeb2 [1]. Among these, a prominent role has been attributed to Snail1 since its expression is widely observed in EMT processes preceding the remaining EMT-TFs; moreover, ectopic Snail1 induces other EMT-TFs such as Zeb1/2 and Snail2 [4], and *Snai1* depletion severely impacts mesoderm formation during embryogenesis [5]. For this reason, Snail1 has been extensively studied as key marker of EMT [6]. Besides this action in epithelial cells, Snail1 is also relevant for fibroblast activation [4], a process also driven in mesenchymal cells for conditions promoting EMT in epithelial cells. Fibroblast activation is required for the generation of cancer-associated fibroblasts (CAFs), a tumor stromal cell with a crucial role in tumor invasion or evasion from the immune system [7]. Without ignoring the contribution of other EMT-TFs to EMT and malignancy, our goal here has been to detail the different mechanisms that control Snail1 expression and function and therefore impact EMT and fibroblast activation.

## 2. Transcription

Snail1 expression was initially studied analyzing its mRNA. First, studies on the control of Snail1 were based on transcription and carried out with human and mouse proximal promoters that present less than 50% of homology. Accordingly, although many similarities are present, transcription factor binding elements described in one of these species cannot automatically be extrapolated to the other. In Table 1, we include a list of the transcriptional factors binding to the promoters of Snail1 genes both in mice and humans.

### 2.1. Murine Snai1 Transcription Regulation

TGFβ was the first factor reported to stimulate *Snai1* transcription and activity of a 900 pb fragment of the *Snai1* proximal promoter [8]. H-Ras transfection is as potent as TGFβ, and both MAPK and PI3K pathways are required for the H-Ras- and TGFβ1-mediated induction of the promoter activity [8]. The role of the canonical TGFβ pathway and Smads in activating *Snai1* promoter in mouse is controversial. The initial observations using a dominant negative form of Smad4 pointed to a Smad4-independent activation [8]; however, in lens epithelial cells, the proximal *Snai1* promoter was activated by TGFβ through the action of Smad2, -3, and -4 [9]. In addition, mice with a specific *Smad2* ablation in keratinocytes show an enhanced EMT during skin cancer formation and progression. In these animals, Smad4 binds to the *Snai1* promoter, and additional *Smad3* or *Smad4* knockdown abrogates Snai1 overexpression [10].

HMGA2 cooperates with the TGFβ/Smad pathway in the activation of *Snai1* gene expression concomitant to an increased binding of Smads to the proximal promoter. While HMGA2 binds to two A/T rich motifs at the −131/−92 region, Smad3 and -4, which physically interact with HMGA2, associate preferentially with the −230/−178 sequence [11]. Myc binding to the *Snai1* promoter is required for rapid *Snai1* activation upon TGFβ stimulation. Accordingly, knockdown of either *c-Myc* or *Smad3/4* in epithelial cells eliminated Snail1 induction by TGFβ [12]. The hepatocyte growth factor (HGF) also activates *Snai1* promoter depending on Myc and Smad4 [12].

The mechanism regulating the expression of the *Snai1* gene has been studied in palatal shelves during the degradation of the midline epithelial seam. To activate expression of *Snai1* in palatal explants, TGFβ3 stimulates binding of Twist1/E47 dimers to the *Snai1* promoter; without E47, Twist1 represses *Snai1* expression [13]. Finally, in the mouse mammary epithelial cells, MMP-3 causes the binding of p65 and cRel NFκB subunits to the *Snai1* promoter, leading to its transcription [14].

### 2.2. Human SNAI1 Transcription Regulation

In humans, *SNAI1* transcription is also controlled by TGFβ and canonical Smads. In many cases, interference with this pathway decreases *SNAI1* mRNA; for instance, in A549 non-small lung cancer cells, the natural dietary flavonoid Kaempferol reverses TGFβ1-mediated *SNAI1* induction by weakening Smad3 binding to the promoter. This is dependent on the selective downregulation of the AKT1-dependent phosphorylation of Smad3 at T179 [15]. In HCCLM3 hepatocellular carcinoma cells, downregulation of AGO1 decreases Smad4 binding to *SNAI1* promoter and reduces its transcription [16]. Liver X receptor α (LXRα) also antagonizes TGFβ since the binding of LXRα to the *SNAI1* promoter prevents that of Smad3/4 [17].

NFκB is another potent stimulator of *SNAI1* transcription and promoter activity. Initial reporter assays with truncated promoters transfected in colon and pancreas cancer cells mapped the NFκB-responsive element to a sequence (−194/−78) located immediately upstream the minimal promoter (−78/+59) [18]. Erythropoietin also increases the binding of p50 and p65 NFκB subunits to the *SNAI1* promoter [19]. Overexpression of v-Akt increases *SNAI1* RNA and promoter activity [18,20]. This Akt effect involves several downstream factors since this protein kinase upregulates *SNAI1* RNA through the activation of NFκB [21] and Smad3 phosphorylation [15].

Another well documented factor that regulates *SNAI1* transcription is STAT3. Chromatin immunoprecipitation assays in cisplatin-resistant atypical teratoid/rhabdoid tumor cells indicated that STAT3 also binds to the *SNAI1* promoter, although in a more distant region than NFκB [22]. STAT3 was found to enhance *SNAI1* induction by TGF-β in cooperation with Ras [23]. In hepatoma cells, phosphorylated STAT3 was also found to bind to the *SNAI1* promoter; inhibition of STAT3 abrogated the hepatitis virus C core-induced Snail1 expression [24]. Additionally, in HCCLM3 hepatocellular carcinoma cells, the isoprenoid antibiotic ascochlorin increased the sensitivity to doxorubicin treatment by directly inhibiting binding of STAT3 to *SNAI1* promoter [25].

Factors downstream MAPK also bind and control *SNAI1* transcription; indeed, the minimal promoter fragment (−78/+59) is dependent on the ERK signaling pathway [18]. Ultraviolet (UV) irradiation transiently induces *SNAI1* expression in human skin and cultured human keratinocytes. Different MAPK pathways (ERK, p38, or JNK) participate in this *SNAI1* regulation, AP-1 sites present in human or mouse promoters and interacting with c-Jun are especially relevant for UV irradiation-increased *SNAI* promoter activity [26]. Osteoblast-derived CXCL5 increases Raf/MEK/ERK activation promoting MSK1 phosphorylation and binding to the *SNAI1* promoter [27]. In human gastric cancer cells, a pre-treatment with N-acetylcysteine attenuated the Helicobacter pylori-induced activation of ERK and *SNAI1* promoter activity [28].

HGF also stimulates Snail1 through MAPK stimulation and through Egr1 that binds to the *SNAI1* promoter [29]. Egr1 is also required for *SNAI1* expression in FGF2-activated cells [30]. Remarkably, Snail1 also binds the *EGR1* promoter and represses the expression of this transcriptional factor [29], demonstrating the existence of a self-inhibitory loop. Other similar loops have been described since Snail itself binds to the *SNAI1* promoter, limiting its own transcription [31]. As discussed in [32], such self-inhibition tends to prevent the aberrant activation of EMT by reducing noise in the system.

Besides these factors, other unrelated proteins, also presented in Table 1, bind and control *SNAI1* transcription [33,34,35,36,37,38,39,40,41,42,43,44,45,46]. Most proteins in this list stimulate *SNAI1* transcription; only metastasis-associated protein 3 (MTA3), aryl hydrocarbon receptor, and CBX8 repress *SNAI1* [44,45,46].

### 2.3. Epigenetic Regulation of the SNAI1 Promoter

*SNAI1* transcription is also regulated by epigenetic modifications at the promoter and enhancer regions. Initial epigenetic studies demonstrated that demethylation of the *Snai1* promoter accompanies its transcription in spindle or dedifferentiated cells and is associated with an increase in acetylated histone H4 [47]. After these studies, many others described methylation and acetylation marks with respect to the *SNAI1* promoter. For instance, in colon cancer cells, the *SNAI1* promoter is regulated by phosphorylated p68 RNA helicase, which induces the dissociation of the HDAC1 from the *SNAI1* promoter and activates its transcription [48]. In breast cancer cells, *SNAI1* is a direct target of JMJD5 and demethylated H3K36me2 [49]. HIV enhances the trimethylation of K4 in H3 at the *SNAI1* promoter site [50]. In nasopharyngeal carcinoma cells, HOPX mediates epigenetic silencing of *SNAI1* transcription through the enhancement of histone H3 K9 deacetylation. In one study, HOPX epigenetically suppressed SRF-dependent *SNAI1* transcription by recruiting histone deacetylase activity [51]. Other examples of epigenetic modifiers include DDX21, recruited to the *SNAI1* promoter together with EZH2 and SUZ12, which increased the trimethylation of H3 on K27 repressing *Snai1* transcription [52]; SETDB1 in MCF7 cells [53]; PDHE1α, which promoted H3K9 acetylation on the *Snai1* promoter to induce transcription and enhance cell motility [54]; MSK1, which enhanced histone H3 acetylation and phosphorylation (S10) at the *SNAI1* promoter [27]; and SATB2, which recruited HDAC1 to silence *SNAI1* transcription [55]. In general, with few exceptions, there is a gap of information on how epigenetic enzymes are activated by the transcription factors binding to the *SNAI1* gene.

Finally, a conserved 3’ region in the *SNAI1* gene acts as an enhancer. Contacting with the constitutively packaged promoter in a poised chromatin structure, this enhancer promotes the transcription associated with the enrichment of H3K4 dimethylation and H3 acetylation, at both the enhancer and the promoter [56]. *SNAI1* transcription is also controlled by a long noncoding RNA, lncRNA-a7, which acts in cis as an enhancer [56]. Accordingly, the deletion of lncRNA-a7 decreases the expression of *SNAI1* mRNA [57].

## 3. mRNA Stability

Many microRNAs (miRNAs) have been shown to negatively correlate with Snail1 levels in a variety of cellular contexts; however, only some of them directly bind and reduce *SNAI1* RNA levels. The earlier miRNA shown to bind to a highly conserved 3’ untranslated region (UTR) in *SNAI1* mRNA was the p53-dependent miR-34 in colon, breast and lung carcinomas. These results unveiled a link between p53, miR-34, and Snail1 in the regulation of cancer cell EMT programs [58]. Preventing miR-34a action by a long non-coding RNA, lncRNA-MUF, which acts as a competing endogenous RNA for miR-34a, leads to Snail1 upregulation and EMT activation [59]. In fact, double-negative feedback loops between the transcription factor Snail1 and the miR-34 family, and the transcription factor ZEB1 and the miR-200 control TGF-β1-induced EMT of MCF10A cells [60]. These loops explain the intermediate phenotypes observed during EMT ([61] and determine the hysteretic and bimodal responses in this transition [62].

The family of miR-30 is also involved in Snail1 repression. During differentiation of tracheal chondrocytes miR-125b and miR-30a/c keep Snail1 at low levels through their binding to the *Snai1* 3’ UTR [63]. In murine hepatocytes, the expression of miR-30 family members is significantly downregulated during TGF-β1-induced EMT, preventing their repressive actions on *Snai1* 3’UTR [64]. In liver fibrosis, miR-30c and miR-193 are a part of the TGF-β-dependent regulatory network controlling Snail1 and extracellular matrix genes [65]. miR-30c, in coordination with miR-26a, and miR-30e-3p also repress *SNAI1* mRNA in other cellular systems [66,67]. Accordingly, miR-30c protects against diabetic nephropathy by suppressing EMT in db/db mice [68].

Several experimental evidences also implicate miR-153 in controlling Snail1 and EMT. In hepatocellular carcinoma, miR-153 inhibits EMT by targeting *SNAI1* [69]. Indeed, the Krüppel-like factor 4 was found to suppress EMT in hepatocellular carcinoma cells in part by inducing miR-153 and repressing Snail1 [70]. Besides these, other reports have described additional miRNAs as also repressing *SNAI1* mRNA in other systems: miR-211-5p in renal cancer [71] and miR-122 in hepatocellular carcinoma [72].

## 4. Translation

Snail1 translation is also regulated both through cap-dependent and -independent mechanisms. Increased expression of Snail1 and a concomitant EMT is promoted by transfection of the Y-box binding protein-1 (YB-1), a protein that activates cap-independent translation of Snail1 mRNA [73]. This rise in Snail1 is antagonized by the cell fate determination factor Dachshund (DACH1) that binds and inactivates YB-1 [74]. Upregulated Snail1 translation caused by YB-1 is dependent on its binding to a putative IRES element contained in the 5’UTR *SNAI1* mRNA. Curiously, other transcriptional factors related to EMT (LEF-1, ZEB2, or HIF1α) are also translationally enriched upon enforced YB-1 expression [73] and contain IRES sequences in their 5’UTRs [75,76,77]. It remains to be established if these factors correspond to proteins activated during specific EMTs triggered by conditions that preclude cap-dependent translation, such as hypoxia.

Snail1 translation is also regulated through eIF4E- and cap-dependent translation. For instance, TGFβ-induced phosphorylation of eIF4E contributes to Snail1 expression during EMT [78]. Accordingly, a chemical antagonist of eIF4E blocks Snail1 mRNA recruitment to the polysomes and EMT [79]. Similar results have also being obtained by the expression of gain-of-function mutants of eIF4E-BP1, a repressor of eIF4E that downregulates Snail1 without affecting its transcription or protein stability [80]. Since eIF4E/eIF4BP1 interaction is disrupted by phosphorylation by TORC1, inhibitors of this modification decrease Snail1 expression. Remarkably, Snail1 also represses eIF4E-BP1 expression by direct binding to the promoter [81], demonstrating the existence of another loop of mutual repression, as has been shown above for Snail1 and miR-34. Accordingly, expression of eiF4E-BP1 and Snail1 is contrary in colorectal tumors [81].

Finally, Snail1 translation is also controlled by methylation of its mRNA. Methytransferase-like 3 (METTL3) modifies a GGAC motif present in the *Snai1* mRNA coding sequence, enhancing its presence in polysomes [82].

## 5. Protein Stability

**Figure 1:**
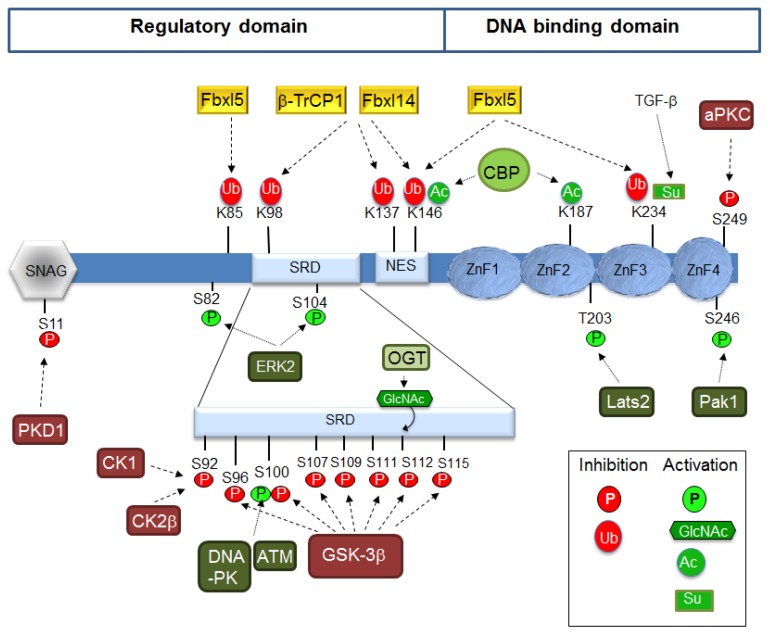
Post-translational modifications controlling Snail1 function. The figure shows a diagram of Snail1 protein with the *N*-terminal regulatory domain and the *C*-terminal DNA-binding domain. The SNAG, SRD, and NES elements are included in the *N*-terminal domain. The indicated covalent modifications of the amino acids are depicted in green or red if they activate or inhibit (respectively) Snail1 function. K48-mediated polyubiquitination is indicated by an oval (Ub); phosphorylation (P) and acetylation (Ac), by circles; glycosylation (NAcGlc), by a hexagon; sumoylation (Su), by a rectangle. The enzymes catalyzing these modifications are also shown when they have been described. Please notice that the phosphorylation of S100 can promote a positive or negative effect on Snail1 function depending on the protein kinase and the context. Only the F-box proteins acting on identified Lysine residues are shown.

Snail1 protein comprises two very well defined parts: the N-terminal or regulatory domain (amino acids 1–148) and the C-terminal or DNA-binding domain [83]. The regulatory domain contains a short sequence in the *N*-terminus, called the SNAG domain with a special relevance for the binding of co-repressors; other relevant regions are the Ser-rich subdomain (SRD) (amino acids 90–120) and the nuclear-export sequence (NES) (amino acids 138–146) [84] (Figure 1). The DNA-binding domain is composed of four zinc fingers (ZnF) of the C2H2 type although ZnF4 does not contain the consensus distance between these residues. A nuclear localization sequence (NLS) is also present in this domain [85].

Snail is a short-lived protein (with a half-life of about 25 min) since it is rapidly ubiquitinated and degraded by the 26S proteasome system [86]. Snail1 ubiquitination involves the participation of several E3 ubiquitin ligases of the multimeric SCF subtype (Skp1-Cullin1-F-box [87]. From about 69 F-box proteins described, eight has been reported to participate in targeting Snail1: FBXW1, FBXW7, FBXL14, FBXL5, FBXO11, FBXO22, FBXO31, and FBXO45. This suggests a highly redundant mechanism of protein degradation to maintain Snail1 levels very low under non-pathological conditions. This seems to be common to other labile substrates with a central role in cancer, such as p53, β-catenin, or c-Myc.

Binding of Snail1 to the F-box module is often associated with Snail1 phosphorylation, although this is not always a prerequisite. The best example of a phosphorylation-dependent interaction is the one with FBXW1, commonly known as β-TrCP1 (β-transducin-repeat containing protein) [88]. SCF-FBXW1/β-TrCP1 recognizes the Snail1 phospho-degron sequence DpS96GxxpS100, a target sequence similarly found in β-catenin and other substrates, located in the SRD and phosphorylated by GSK-3β [89] (Figure 1). GSK-3β action requires the previous priming of Snail1 S92 by CK1ε or CK2β [90,91]. Other E3 ligases also require previous phosphorylation, such as SCF-FBXO11, which requires phosphorylation by the protein kinase D1 (PKD1) of Snail1-S11 in the SNAG domain [92]; however, according to other authors, it may also occur independently on phosphorylation [93]. Snail1 is degraded by the FBXW7 tumor suppressor [94,95]. Although FBXW7-mediated degradation will probably require Snail1 phosphorylation, as shown for ZEB2 [96] and many other substrates (cyclin E, Notch, c-Jun, c-Myc and mTOR) [97], this point has not been formally proven in the case of Snail1. Other F-box proteins have also been proposed as phosphorylation-dependent ubiquitin ligases for Snail1 such as SCF-FBXO22 and SCF-FBXO31 in mammary and gastric carcinomas, respectively [98,99].

The fact that in many cells Snail1 degradation is independent on GSK-3β suggested the participation of other E3s; accordingly, SCF-FBXL14 was identified as a potent, phosphorylation-independent Snail1 E3 ligase [100]. FBXL14 and β-TrCP1 redundantly modify the same group of Snail1 lysines (K98, K137, and K146) [100]. FBXL14 seems to have a central role in EMT since it also acts on other EMT-TFs and targets Snail2, Twist1, and Zeb2 [101]. SCF-FBXL14 is transcriptionally repressed during hypoxia, leading to Snail1 stabilization [100]. Importantly, hypoxia activates a full EMT program with the concomitant induction of Snail1, Twist1, and Zeb2 [102,103]. Recently, LKB1 protein has been shown to regulate FBXL14-Snail1 interaction by increasing their affinity [104].

An independent shRNA screening identified SCF-FBXL5 as a nuclear Snail1 E3 ubiquitin ligase binding to Snail1 C-terminal and targeting lysines 85, 146, and 234 [105]. Besides promoting degradation, Snail1-K234 ubiquitination by FBXL5 also decreases Snail1 interaction with the DNA. Curiously, FBXL5-mediated degradation is blocked after nuclear export inhibition, suggesting that the cytosolic relocation of ubiquitinated Snail1 is required in order to be efficiently degraded. FBXL5 protein stability requires iron and oxygen that bind to its N-terminal hemerythrin domain [106,107] and it is decreased by hypoxia [107] and by γ-irradiation (IR) [105]. FBXL5 suppresses invasion of gastric cancer cells by reducing the levels of Snail1 [108].

Finally, another E3 ligase acting on Snail1 is FBXO45 (F-box/SPRY domain–containing protein 1). Besides Snail1, this enzyme targets Snail2, Zeb1/2, and Twist1 [109]. In contrast to the other Snail1 E3 ligases, FBXO45 does not form an SCF complex [110].

It is remarkable that most FBX proteins controlling Snail1 stability are regulated by miRNAs: miR-17/20a controls FBXL14 [111], miR-27a targets FBXO45 [109], and FBXL5 mRNA levels are negatively regulated by miR-1306-3p; therefore, miR-1306-3p expression results in increased Snail1 protein stability [112].

As stated before, Snail1 degradation is intimately related with its phosphorylation status [86]. For this reason, Snail1 C-terminal domain dephosphorylation by small phosphatases promotes Snail1 stabilization [113,114]. Recently, the protein tyrosine phosphatase PTEN has been shown to change its tyrosine phosphatase activity after MEX3C-catalyzed K27-linked polyubiquitination triggered by high glucose, TGFβ or IL-6; K27-polyUb PTEN dephosphorylates the phosphoserine/threonine of several proteins involved in EMT, including S96 in Snail1, leading to its accumulation [115]. Snail1 stabilization is also triggered by TNF-α during inflammation and is mediated by the COP9 signalosome 2 protein (CSN2), which blocks the phosphorylation and ubiquitination of Snail1 by disrupting its binding to GSK-3β and β-TrCP1 [116]. However, phosphorylation is not always linked to degradation, and, intriguingly, some kinases may modify residues involved in protein instability to induce the contrary effect, Snail1 stabilization. This is the case for ATM that phosphorylates S100 increasing Snail1 half-life [117]; however this residue has also been related to GSK-3β-induced Snail1degradation [89]. This opposed regulation may be dependent on the different interaction of phosphorylated Snail1 with specific factors present in the nucleus or in the cytosol.

Other Snail1 stabilizing phosphorylations modify specific residues located in the *C*-terminal domain (Figure 1). This is the case for Lats2 kinase induced by TGFβ, which phosphorylates Snail1 on T203 [118] or the p21-activated kinase 1 (PAK1) that acts on S246 [119]. Curiously, whereas modification of this residue stabilizes Snail1, that of S249 by PAR-atypical protein kinase C (aPKC) leads to its degradation [120].

Interestingly, gamma-irradiation and DNA damage promote Snail1 expression and protein stabilization. This effect is mediated through the activation of PAK1 phosphorylating S246 [119] and by ATM and DNA-dependent protein kinase (DNA-PK) that modify S100 [117,121], suggesting the convergence of kinases regulated by cell stress. Other kinases inducing stabilization phosphorylate residues from the Snail1 N-terminal domain as ERK2, which acts on S82 and S104 after being activated by the collagen receptor known as Discoidin domain receptor 2 [122].

Ubiquitination can also promote an increase in Snail1 half-life when catalyzed by Pellino-1, which promotes Snail1 K63-mediated polyubiquitination [123], or by A20 that multi-monoubiquitinates Snail1 [124]. Other, less-studied post transcriptional modifications (PTMs), which also affect Snal1 stability, are polyADP-ribosylation (PARylation) [125] and glycosylation [126]. Snail1 modification by β-N-acetylglucosamine (O-GlcNAc) is triggered by high-glucose levels and has been mapped to S112, preventing phosphorylation by GSK-3β [126]. Snail1 is also modified by sumoylation of K234, a PTM enhanced by TGFβ that controlsSnail1 nuclear retention and cell invasion [127]. Finally, Snail1 is also acetylated by the CREB-binding protein (CBP) at lysines 146 and 187, a modification crucial for its transcriptional activity [128], as discussed below. It has been reported that Snail acetylation also enhances its stability by inhibiting phosphorylation and ubiquitination [129].

E3-dependent protein ubiquitination can be reverted by deubiquitinating enzymes (deubiquitinases, or DUBs), which play a decisive role in substrate stabilization [130]. Snail1 interacts and is deubiquitinated by DUB3 (also known as USP17L2) [131,132] and by USP27X [133]. These two DUBs are induced by cytokines: DUB3 by IL-6 [132] and USP27X by TGFβ [133]. Recently, other DUBs (OTUB1, PSMD14, USP11, USP26, and USP47) have been reported to promote Snail1 deubiquitination [134,135,136,137,138].

## 6. Subcellular Localization

Snail1 transcriptional action requires its accumulation in the nucleus, which seems to be the consequence of inhibited export. Besides being required for function, retention in the nucleus also indirectly controls Snail1 stability, since the most active Snail1 ligases (βTrCP1 and Fbxl14) are located in the cytosol [87]. Nuclear import is mediated by a C-terminus conserved NLS recognized by importins [85]. Nuclear export requires phosphorylation by GSK-3β on residues S104 and S107 to S119; this uncovers the NES (aa 132–143) that binds to Crm1 (Exportin-1) [84,89,139]. Alternatively, Snail1 ubiquitinylation by FBXL5, besides interfering with DNA-binding, facilitates nuclear export and Snail1 degradation in the cytosol [105].

Snail1 nuclear retention is triggered by GSK-3β inhibition. This is accomplished after Wnt stimulation that promotes the Axin2/GSK-3β nuclear export; therefore, phosphorylation-induced Snail1 traffic to the cytosol is blocked [140]. Akt phosphorylation of S9 in GSK-3β also inhibits this enzyme [89]; therefore, pathways activating Akt, such as TNFα, Wnt, and Notch, promote Snail1 nuclear retention [141]. Snail1 nuclear export is also prevented by dephosphorylation [113] or glycosylation, incompatible with the phosphorylation of S112 [126] (see Figure 1). Indirectly, inhibition of priming of GSK-3β phosphorylation also inhibits nuclear export [89,90,142,143]. Other protein kinases stabilizing Snail1 (Lats2, PAK1, or ERK2) also promote their effects enhancing Snail1 nuclear retention and preventing cytosolic degradation [118,119,122]. The mechanisms are not fully understood but may involve the participation of nuclear chaperones such as HSP90 or HSP27, which inhibit the binding of Snail1 to the Crm1 nuclear exporter [117,144]. Recently, the mitogen-activated sumoylation of nuclear Flotilin-1 has been reported to raise its interaction with Snail1 in this compartment and increase its stability [145].

## 7. Post-Translational Modifications Controlling Interaction with Co-Repressors and Co-Activators

**Figure 2:**
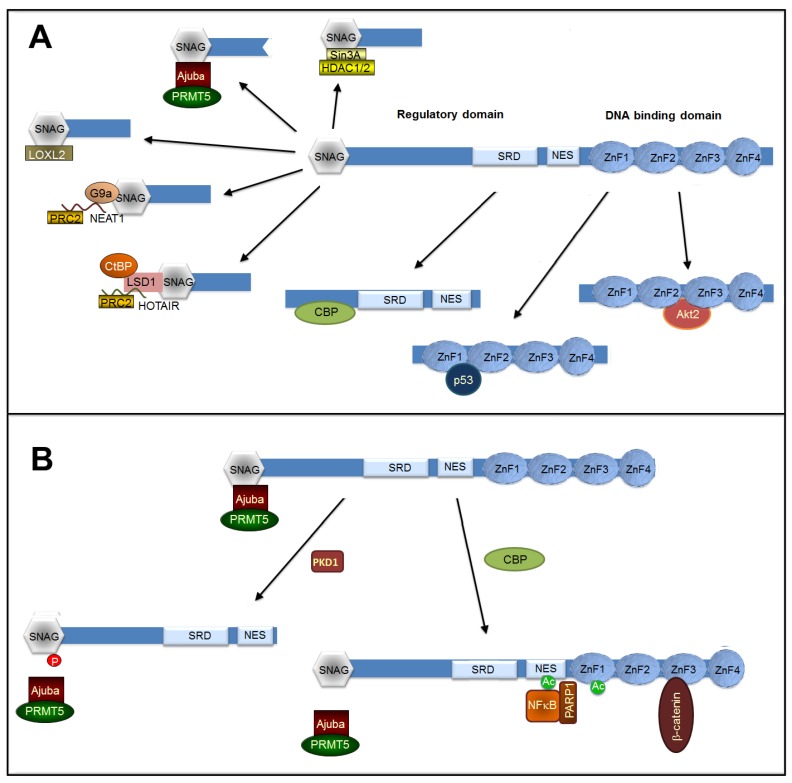
Transcriptional cofactors required for Snail1 function. The figure shows a diagram of Snail1 protein with the different corepressors interacting with the SNAG domain (**A**). The binding sites for CBP (required for Snail1-induced activation or mesenchymal genes), p53, and Akt2 are also shown. Other factors also interacting with Snail1 but with still uncharacterized sequences are not shown. In panel (**B**), the effect of PKD1-induced phosphorylation of S11 in the SNAG sequence on the interaction of Ajuba complex is presented. Moreover, this panel also illustrates the switch in Snail1 function promoted by the CBP-catalyzed phosphorylation of K146 and K187 that disrupts the association with Ajuba and facilitates binding to the p65/NFκB complex and presumably also to β-catenin. The Snail1 element interacting with NFκB has not been characterized; binding to β-catenin has been allocated to the C-terminal domain, likely to ZnF3 and -4 (see text).

Besides interacting with proteins involved in its stability or subcellular localization, Snail1 binds to other factors required for its transcriptional function. Although initially described as a repressor, several reports have determined that Snail1 also actively participates on gene transcription through its binding to mesenchymal promoters [128,146]. This different activity of Snail1 protein as transcriptional repressor or activator is controlled through its binding to different proteins, interactions that are also sensitive to post-translational modifications. We described here some of the cofactors required for these two Snail1 functions. A scheme of the binding of these different cofactors is presented in Figure 2A.

### 7.1. Snail1 Binding to Co-Repressors

The capability of Snail1 to inhibit gene transcription requires its interaction with specific E-boxes presenting a core sequence of 5’-CACCTG-3’ (or inverse, 5’-CAGGTG-3’). This binding is mediated by the C-terminal Snail1 domain containing four zinc-fingers. Interestingly, the presence of a Smad-binding element closer to the E-box (about 100 bp) enhances Snail1 repression since Snail1 interacts with the Smad3/Smad4 complex [147]. This can indirectly potentiate the binding of Zeb1 and -2 proteins (also known as Tcf8 and Sip1, respectively) to adjacent E-boxes, since these two factors also associate with Smads [148,149], potentiating and temporally extending the Snail1 repression of epithelial genes.

In contrast to the Drosophila orthologue [150], Snail1 does not contain a binding site for the CtBP co-repressor. Instead, most of the cofactors involved in gene silencing interact with the short SNAG sequence (13 amino acids) placed in the very *N*-terminal end. For instance, Snail1 associates with the histone deacetylase (HDAC) complex Sin3a/HDAC1/HDAC2; this binding is dependent on the integrity of the SNAG domain [151]. It is unclear if this association is direct or mediated by LIM proteins, a family of proteins that mediate nuclear signaling events. Accordingly, the LIM proteins Ajuba and FHL2 interact with Snail1 and promote E-cadherin repression [152,153,154]. Besides HDACs, Ajuba also participates in the recruitment of protein arginine methyltransferase 5 (PMRT5), another histone modifier related to gene repression [155]. Snail1 phosphorylation in S11 by PKD1, besides promoting other negative effects on Snail1 function (see Section 5), prevents Snail1 repression by disrupting the interaction with Ajuba [142] (Figure 2B). It is remarkable that Ajuba, like β-catenin, is detected in cadherin-dependent junctions [156], representing an element of the cross-talk between cell–cell contacts, Snail1 function, and EMT (see also below).

Besides HDAC1/2 and PRMT5, other epigenetic regulators such as Polycomb repressive complex 2 (PRC2) are also necessary for Snail1-dependent E-cadherin repression [157]. PRC2 binding to Snail1 also requires the SNAG domain [157] and is associated with the formation of a complex also involving HDAC1 and -2 [158]. Since the PRC2 subunit EzH2 interacts with long non-coding RNAs (lncRNAs) [159], it has been proposed that the lncRNA HOTAIR mediates the interaction of Snail1 to EzH2 through the physical interaction of this lncRNA with both proteins [160]. However, it is possible that it is not Snail1 by itself that binds to HOTAIR but a Snail1-associated factor. A good candidate for this is the Lysine-specific methylase 1 (LSD1)/CoREST/REST complex since it interacts with HOTAIR [159] and to Snail1 through a mechanism in which the SNAG Snail1 domain mimics the histone tail and binds to the active site of LSD1 [161]. Moreover, since LSD1 also associates with CtBP1 [162], it provides the molecular connection between CtBP and the Snail1-repression complex, an association that in Drosophila Snail1 is accomplished by a direct Snail1-CtBP binding [150].

Another co-repressor binding to the Snail1 SNAG sequence is G9a, which forms part of the G9a histone methyltransferase/DNA methyltransferases complex [163]. This complex also interacts with another lncRNA, NEAT1, and is required for the NEAT1- and Snail1-dependent E-cadherin repression [164]; therefore, it is possible that G9a and NEAT1 might also participate in PRC2 recruitment to the Snail1 repressive complex.

Snail1 SNAG domain also binds to Lysyl oxidase-like 2 (LOXL2) [165]. Although it is still a matter of discussion, since for some authors the activity of this enzyme is not relevant in EMT [166], LOXL2 acts as an epigenetic modifier and participates in Snail1-induced gene repression by oxidizing and demethylating K4 in histone 3 [167] and TAF10, blocking TFIID-dependent gene transcription [168]. It is still unknown if, as is the case with Ajuba, binding of G9a, PRC2, and LOXL2 is also blocked by PKD1-dependent Snail1 phosphorylation in S11 or by acetylation (see below).

### 7.2. Snail1 Binding to Transcriptional Activators

Although Snail1 has been extensively studied as a transcriptional repressor, increasing evidence indicates that it also directly activates transcription. Binding of Snail1 to activated promoters of several genes has been reported [146,169,170,171]. In Drosophila, Snail positively modulates the transcriptional activation of target genes involved in the development through binding to active enhancers [172]. A kinetic study of Snail1 promoter binding during TGFβ-induced EMT has revealed that an initial phase of Snail1 association with repressed promoters, such as *CDH1* (E-cadherin), is followed by the later interaction with mesenchymal genes, such as *FN1* (fibronectin), concomitant with the transcriptional activation of these genes [146]. Whereas repression is dependent on canonical Snail1-binding 5’-CACCTG-3’ boxes in the promoter, activation is not and is produced by a Snail1 association with an NFκB/PARP1 complex [146]. Snail1 also interacts with β-catenin stimulating β-catenin-mediated transcription [173]. In both cases, the association does not require the SNAG domain; the binding site in Snail1 has been characterized and corresponds to the C-terminal domain only for β-catenin [173]. Recently, a proposed Snail1-responsive motif, 5’-TCACA-3’, has been identified in the promoters of ZEB1, MMP9, and p15INK4, genes activated by Snail1 in collaboration with EGR and SP1 [171]. This Snail1 switch from acting as a transcriptional repressor to an activator is dependent on its interaction with CBP, which acetylates K146 and K187 [128]. Accordingly, CBP and the co-repressors Ajuba and Sin3a are not present in the same Snail1 complexes and ectopic expression of CBP prevents the interaction of Snail1 and these co-repressors [128]. At present, it is not known how modification of these two Lysine residues might disrupt the association of repressors to the SNAG N-terminal sequence and facilitate the binding to co-activators (Figure 2B).

### 7.3. Other

As previously indicated (see Section 3), Snail1 and p53 are mutual antagonists, and p53 de-stabilizes *SNAI1* mRNA by activating miR-34 [58]. Snail1 also represses p53 function. The mechanism of this inhibition is still a matter of discussion: according to some authors, Snail1 and p53 interact [174]; as a consequence, p53 is secreted to the cellular medium and degraded [175]. Other authors have observed the presence of Snail1 and p53 in a complex with HDAC1, which promotes p53 deacetylation and further degradation by the proteasome [176]. However, in other cells, for instance in mesenchymal stem cells, p53 levels are not different in Snail1 KO or control cells [177]. It is unknown if the Snail1 interaction with p53, which requires the two first zinc-fingers in the C-terminal domain (aa 154–208) according to some authors [176] or the middle region (aa 91–112) according to others [174], is controlled by specific PTMs, which might explain the contradictory results observed in different cells.

Snail1 also binds to Akt2 [178], a protein kinase tightly associated with Snail1 function, since it is involved in Snail1 transcription and protein stabilization (see above) and is activated upon Snail1 expression [179,180]. Association with Snail1 enhances Akt activity on T45 in histone H3, a modification associated with transcription termination after DNA damage [181]. Since Snail1 is also upregulated by this insult [153] Snail1–Akt2 binding might contribute to the higher resistance to DNA damaging agents detected in Snail1-expressing cells.

## 8. Conclusions and Future Perspectives

Since Snail1 was characterized in 2000 as a transcriptional repressor of *CDH1* and an inducer of EMT and invasion in tumor cells [83,182], it has been the subject of study for many cancer biologists. Moreover, the implications of Snail1 and EMT in drug resistance, in the acquisition of cancer stem properties, and in other traits involved in tumor development have further fostered this interest. The analysis of Snail1 function has revealed a multilevel control that impinges on all the processes required for protein expression; moreover, multiple PTMs of this protein activate or inhibit Snail1 function. This multiple control has been frequently ignored, and the expression of Snail1 is usually determined only on the basis of its RNA levels, which do not necessarily correlate with protein or Snail1 function, as shown above. Moreover, several aspects of Snail1 action have yet to be clarified, especially those related to its role as a transcriptional activator of mesenchymal genes. It is likely that future research on Snail1 will clarify these issues and better explain Snail1 function in tumoral cells.

## Figures and Tables

**Table 1:** Transcription factors binding to the *Snai1* or *SNAI1* promoter.

Binding Factor	Cell Line or Tissue	References
Smads	Lens epithelial cells, keratinocytes and lung and liver cancer cells	[9,10,15,16,17]
HMGA	Mammary and liver epithelial cells and fibroblasts	[11,12]
NFκB	Mammary epithelial cells and breast, colon, and pancreas tumor cells	[14,18,19]
STAT3	Atypical teratoid/rhabdoid tumor cells, liver cancer, and pancreatic epithelial cells	[22,23,24]
Twist/E47	Palatal shelves	[13]
AP1	Skin keratinocytes	[26]
ELK1/MSK1	Breast tumor cells	[27]
Egr1	Stomach, esophagus, and liver cancer cells, kidney epithelial cells, and embryonic stem cells	[29,30]
Snail1	Colon and pancreas cancer cells and fibroblasts	[31]
Forkhead box M1	Lung adenocarcinoma cells and endothelial cells	[33,34]
PARP1	Breast epithelial cells and prostate cancer cells	[35]
Polyomavirus-enhancer activator 3	Lung and ovarian cancer cells	[36]
MUC1	Renal carcinoma cells	[37]
P4R/EGFR	Liver cancer cells	[38]
COUP-TFII	Colon cancer cells	[39]
SP1	Cholangiocarcinoma cell lines	[40]
HIF1α	Hepatocellular carcinoma	[41]
Estrogen receptor	Breast cancer cells	[42]
Wilms’ tumor-1	Epicardial cells	[43]
MTA3	Breast cancer cells	[44]
Aryl Hydrocarbon receptor	Gastric carcinoma cells	[45]
CBX8	Esophagus cancer cells	[46]

Those commented in the text are in bold.
